# The need to include animal protection in public health policies

**DOI:** 10.1057/jphp.2013.29

**Published:** 2013-06-27

**Authors:** Aysha Akhtar

**Affiliations:** 1 Oxford Centre for Animal Ethics, 91 Iffley Road, Oxford, OX4 1EG England UK; 2 Office of Counterterrorism and Emerging Threats, FDA, 10903 New Hampshire Avenue, Silver Spring, 20993 MD USA

**Keywords:** animals, public health, animal treatment, emerging infectious diseases, medical research, domestic violence

## Abstract

Many critical public health issues require non-traditional approaches. Although many novel strategies are used, one approach not widely applied involves improving the treatment of animals. Emerging infectious diseases are pressing public health challenges that could benefit from improving the treatment of animals. Other human health issues, that overlap with animal treatment issues, and that warrant further exploration, are medical research and domestic violence. The diverse nature of these health issues and their connection with animal treatment suggest that there may be other similar intersections. Public health would benefit by including the treatment of animals as a topic of study and policy development.

## Introduction

Environmental hazards, stagnation of drug development, climate change, human population growth, emerging infectious diseases (EIDs), world hunger, and violence are among our most urgent public health concerns. Their multifactorial roots necessitate strategies that go well beyond the traditional health sector. One strategy that has been largely neglected and that can help address several of these issues involves improving the treatment of non-human animals (hereafter ‘animals’). I briefly describe how EIDs are connected with the treatment of animals and discuss why this connection needs to be addressed in public health strategies. I describe other areas of exploration, including issues concerning domestic violence and medical research.

## Emerging Infectious Diseases

Since 1980, more than 35 infectious diseases have emerged and 87 human pathogens have been discovered, an average rate of about three new pathogens each year.^1, 2^ Of the 175 human pathogens that have been classified as emerging or re-emerging, three-fourths come from animals.^3^ Several factors contribute to the increase in EIDs, including environmental changes and human population growth. Changes in animal agriculture and growth of the wildlife trade also contribute to the increase in emerging infections.

### Animal farming

Each year, more than 64 billion animals are raised and killed for food globally.^4^ Largely because of increased demand for animal products, intensive animal operations have mostly replaced traditional farming practices worldwide, particularly for pigs and poultry.^5^ The stress and distress associated with these new farming conditions heightens animals’ vulnerability to disease. A report published by the Pew Commission highlights how close contact of animals facilitates the exchange and evolution of pathogens and how stress induced by dense confinement increases the likelihood of infection and illness in animals.^6^ Pathogens circulating in these animal populations may lead to human illnesses.

For centuries, the evolution of the influenza virus had remained relatively stable.^7, 8, 9^ In recent years, however, the virus has undergone an evolutionary surge, with new variants emerging rapidly. The intensive confinement of animals is shown to be a major contributor to this surge.^9, 10^ Highly pathogenic avian influenza (HPAI) H5N1, first isolated in the Guangdong Province of China in 1996, is one of the most notable pathogens to appear recently.^11, 12^

Most avian influenzas begin as mildly pathogenic viruses.^13^ Once they enter domestic bird populations, they may rapidly mutate into highly pathogenic viruses. Research demonstrates that after circulation in domestic birds for very short periods, low-pathogenic avian influenza (LPAI) viruses can mutate into HPAIs.^14^ Since 1990, outbreaks of HPAI virus subtypes have become more common in farmed birds compared with before 1990.^8, 9, 14^ Intensive confinement of birds facilitates both the increasing frequency and scale of these outbreaks.^9, 10^ According to the World Organization for Animal Health of the Food and Agriculture Organization, two lessons should be learned from these prior outbreaks.^15^ First, that if LPAI viruses are allowed to spread among farmed birds, they can eventually mutate into HPAI viruses; and second, that densely confining birds considerably increases their vulnerability to infectious diseases.

Thus far, H5N1 has shown limited human-to-human spread. Pigs, however, may help H5N1 gain the ability to widely infect humans.^16^ Pigs are highly susceptible to both avian and human influenza A viruses.^17, 18^ Until recently, only sporadic reports described pigs infected with H5N1, but a study published in 2010 reveals that between 2005 and 2007, 7.4 per cent of 700 pigs tested in Indonesia carried H5N1, even though they lacked influenza-like symptoms.^8, 19^ One viral isolate had acquired the ability to recognize a cell receptor present in the noses of both pigs and humans. This change might allow it to spread easily from pigs to humans and then among humans. The pigs may have been initially infected from nearby chicken farms, but there was also evidence of pig-to-pig transmission.

### Wildlife trade

The transporting of animals across the globe also influences the emergence and spread of infectious diseases. Millions of animals annually are caught in the wild or bred in captivity for the *wildlife trade*. They are traded as exotic pets, entertainment, food, skins, medicinal objects, and for biomedical research. Trade, both international and within countries, has increased rapidly worldwide.^20, 21^

Few laws protect animals from harm during any phase of trade.^22, 23^ Where regulations exist, they are rarely enforced or the penalties are so minor that they provide almost no deterrent.^22^ As a result, overcrowding, exposure to extreme temperatures, unsanitary conditions, and animal diseases are common.

The trade creates ideal conditions for pathogens to multiply. Of all contributors to the emergence of zoonotic pathogens (such as ecological factors and behavior), ‘species-jumping’ events may be among the most important.^24^ They expand the range of viable hosts. Holding different populations of animals together, particularly sick and stressed animals, may result in the spread of new pathogens. A 2003 outbreak of monkeypox in the United States occurred after a shipment of infected African Gambian rats was sold to pet dealers, one of whom housed the rats with prairie dogs.^25, 26^ The prairie dogs contracted monkeypox, then were sold as pets, and transmitted the pathogen to 71 people.

Severe Acute Respiratory Syndrome (SARS) probably emerged from the wildlife trade in Guangdong Province, China.^27, 28^ Chinese authorities slaughtered thousands of civets, small arboreal mammals traded for their musk-producing glands, because they were suspected to be the source of the pathogen. SARS is now believed to have originated from large fruit bats.^29, 30^ Bats had been captured in the wild and traded live in Chinese markets. At some point in the wildlife supply chain, infected bats were likely brought into contact with susceptible hosts, such as civets, in whom the virus amplified. The intermingling of species established a cycle in which susceptible animals and humans could become infected. After the SARS epidemics subsided, an editorial in the *American Journal of Public Health* made this observation: ‘The concentration of animals, their overlapping sojourns in the markets (allowing disease to spread through vast numbers of animals), and their interactions with humans (facilitating human infection) make these markets ripe for zoonoses. Once an epidemic starts among these animals, it can spread to animals reared in less cruel conditions’.^31^

Butchering and eating of non-human primates through the *bushmeat* trade probably led to the emergence of HIV.^32, 33^ Similarly, Ebola virus has been traced back to the trade in non-human primates, bats, forest antelopes, rodents, and shrews.^34, 35, 36^ The Nipah virus, first identified in the Sungai Nipah New Village in Malaysia, spread throughout the region through the trucking of infected pigs.^37^

## Other Connections

Two other connections between human health and the treatment of animals warrant further exploration: medical research and interpersonal violence. More than 115 million animals are used each year in experiments or to supply the biomedical industry.^38^ Although the topic of animal research has not traditionally been viewed as a public health issue, its use is inextricably tied to the safety and efficacy of medical therapeutics, that play an integral role in many public health applications, including those intended to combat EIDs. Successful anti-infectious disease campaigns, for example, often rely on the availability of safe and effective vaccines and therapeutics, that in turn, depend on reliable and predictive pre-clinical research. A new study found that the mouse models used extensively to study human inflammatory diseases, including infectious diseases, only poorly mimic the human condition.^39^ This calls into question the reliability of mouse models to identify, develop, and test drug and vaccine candidates.^39^

Indeed, because of apparent stagnation in the invention of useful drugs, leaders in the biotechnology and pharmaceutical industries have argued that poor predictability of animal testing is among the chief challenges facing the drug discovery community.^40^ The stresses induced in the laboratory can affect animals’ physiology and perturb study results.^41, 42^ This begs the question: How relevant is animal testing for predicting human health outcomes?^43, 44, 45, 46, 47, 48^ Researchers need to develop human-based testing methods to better identify and predict the safety and efficacy of potential pharmaceutics.

The establishment of non-animal testing methods to replace animal use remains underdeveloped, with too few resources devoted to the endeavor.^49, 50, 51^ Investment in alternative testing methods fall far short of investments in animal testing.^51^ Making predictive human-based testing models a higher priority would improve medical research and reduce the numbers of animals used in harmful experiments.

Domestic violence has become a public health urgency worldwide. As perpetrators of violence usually harm all those physically weaker than themselves, abuse of animals occurs frequently in conjunction with human abuse.^52, 53, 54^ Domestic service and child protection organizations, such as the UK’s National Society for the Prevention of Cruelty to Children, have called animal abuse a ‘red flag’ for other forms of violence.^54, 55^ Merging anti-abuse strategies – human and animal – can improve detection of all forms of violence.^56^ Unfortunately, health agencies do not give the human–animal violence link sufficient weight in public health policy. Neither the World Health Organization nor the US Centers for Disease Control and Prevention, for example, emphasize the connection.

## Limitations of Current Public Heath Strategies – Suggestions to Move Forward

The examples above demonstrate a connection between human health and the treatment of animals. Awareness of this link grows. The One Health Initiative now fosters collaboration between veterinary and human health professionals, and works, for example, to improve detection of EIDS.^57^ Although a positive step, this initiative misses a vital point that many EIDS are due at least partly to the treatment of animals. Yet, animal treatment is largely absent from public health dialogue. Current strategic policies, which do not fundamentally address animal treatment, are off the mark.

Interventions against EIDs target surveillance and inspection activities, animal vaccination campaigns, and culling, but do not confront an underlying cause, the poor treatment of animals. Surveillance is constrained by our infrastructure plus scientific limitations. Relying on inspection of shipments remains impractical. Gerson and colleagues estimated that only 2 per cent of the more than 12 million commercial shipments into Canada annually are physically inspected.^58^ Pathogens that cause little or no overt signs of illnesses in animals will likely go undetected. The H7N9 avian influenza virus, for example, does not cause apparent illness among farmed birds, thus silent spread puts humans at risk.^59^ Trying to predict which virus subtypes circulating among animals that will pose dangers to humans is extremely difficult.^60^

Culling and vaccination campaigns may in the short term quell an epidemic, but they have frequently failed to provide lasting solutions. More than 100 million birds were killed throughout Asia in an effort to thwart the spread of H5N1.^61^ However, the next wave of H5N1 re-established itself in the same countries and spread to new ones. Poultry vaccination programs in Asia and Egypt did not prevent the re-emergence and spread of H5N1.^62, 63^ Indeed, vaccination may spur the evolution of the virus.^64, 65^ Whether vaccination programs in China lead to greater genetic diversity of the H5N1 virus and contribute to current strains is one of many questions to consider.^66^

Moreover, was a low-pathogenic precursor to H5N1 from wild birds introduced to poultry populations through household or ‘backyard’ flocks? Or was a precursor introduced through commercial confinement operations? Although backyard flocks may serve as conduits that transfer avian influenza viruses to domestic bird populations, their contribution to the emergence and spread of H5N1 may have been overestimated. In an analysis of a 2004 poultry outbreak of H5N1 in Thailand, Graham and colleagues found the likelihood of outbreaks to be far greater in large-scale commercial operations than in backyard flocks.^67^ Additionally, there are numerous routes by which pathogens can be introduced to commercial operations. Studies in Canada, the Netherlands, and Denmark reached similar conclusions.^68^

The discovery of H7N9 suggests that current mitigation efforts are failing to prevent the emergence of new, dangerous strains. Ultimately, strategies will have the greatest chance of success in preventing further evolution of avian influenza and other pathogens if they confront the underlying causes of pathogen emergence, some of which are rooted in the poor treatment of animals.

Direct benefits to human health are possible if efforts to improve the treatment of animals are routinely incorporated into public health policy and strategies. If, to benefit animals, we focus on reducing the number of animals confined in industrial farms and traded worldwide, opportunities for the emergence and spread of new pathogens would decrease and benefit people. Plant-based diets can reduce the number of animals confined in industrial farms and further decrease emergence of zoonotic pathogens. Such diets also confer environmental and personal health benefits.^5, 22^

Might animal protection organizations join public health colleagues to forge a public campaign against the keeping of exotic animals as pets and for entertainment? Public health agencies and animal protection organizations should work with policymakers to enact restrictions on wildlife importation. Reduced use of animals in experiments deserves emphasis by government institutions that fund research.^22^ Finally, if there were greater coordination on animal protection between public health, veterinary and social services, together we might increase detection of all forms of violence and thwart future acts of violence.^56^

## Conclusion

The emergence of many recent pathogens can be attributed, directly or indirectly, to the intensive confinement of animals raised for food and the poor treatment of animals appropriated for the wildlife trade. The strategies currently used to address EIDs would be much improved if efforts to improve the treatment of animals were integrated into public health policies.

Studying the connection between domestic violence and animal mistreatment can surely help control both problems. Strategies combating animal cruelty may increase detection and prevention of violence against humans. Similarly, critically assessing the value of the use of animals in research is likely to benefit animals and improve research. We should strive to replace animal experiments with more predictive human-based testing methods. With further study, other connections between human health and animal treatment are likely to be discovered.

By discussing poor animal treatment, I do not intend to lay blame, but rather to offer suggestions for improved public health strategies. By ignoring the overlaps between human health and the treatment of animals, we may fail to see solutions to critical public health problems. If we do not recognize this connection, where it exists, opportunities to tackle important health issues may be lost.
